# Romosozumab Versus Teriparatide for the Treatment of Postmenopausal Osteoporosis: An Overview of Systematic Reviews With Direct and Indirect Meta‐Analyses

**DOI:** 10.1111/1756-185x.70658

**Published:** 2026-04-17

**Authors:** Tayna Felicissimo Gomes de Souza Bandeira, Patricia Melo Aguiar, Cid Manso de Mello Vianna, Gabriela Bittencourt Gonzalez Mosegui, Tácio de Mendonça Lima

**Affiliations:** ^1^ Faculty of Pharmacy Fluminense Federal University Niteroi RJ Brazil; ^2^ Department of Pharmacy, Faculty of Pharmaceutical Sciences University of Sao Paulo Sao Paulo SP Brazil; ^3^ Institute of Social Medicine State University of Rio de Janeiro (UERJ) Rio de Janeiro RJ Brazil; ^4^ Institute of Collective Health Fluminense Federal University Niteroi RJ Brazil; ^5^ Department of Pharmacy and Pharmaceutical Administration Fluminense Federal University Niteroi RJ Brazil

**Keywords:** overview, postmenopausal osteoporosis, romosozumab, systematic review, teriparatide

## Abstract

**Aim:**

To summarize the evidence of efficacy and safety of romosozumab compared to teriparatide for postmenopausal osteoporosis.

**Methods:**

A literature search was performed in Medline, the Cochrane Library, CRD, and LILACS until November 2023. We included systematic reviews with meta‐analyses on the use of romosozumab compared to teriparatide in women with postmenopausal osteoporosis. Two authors performed study selection, data extraction, and quality assessment using AMSTAR‐2 and GRADE tools.

**Results:**

A total of 725 records were identified, and 13 studies fully met the eligibility criteria. Regarding efficacy, romosozumab did not show a significant difference in risk of falls (12/24 months), vertebral fractures (12/36 months), non‐vertebral fractures (12/36 months), and hip fractures compared to teriparatide. For the safety profile, romosozumab did not show significant change compared to teriparatide for serious adverse events (12/26 months) and composite cardiovascular outcomes (3PMACE and 4PMACE). The overall quality of evidence in the reviews ranged from very low to moderate, primarily due to indirectness and imprecision in the outcomes. Additionally, the reviews were classified as having “low” or “critically low” methodological quality.

**Conclusion:**

Romosozumab demonstrated efficacy and safety similar to teriparatide. However, this overview revealed substantial overlap among primary studies, with most outcomes assessed through indirect comparisons and poor methodological quality. Future research should confirm these findings.

## Key Points

- Romosozumab demonstrated comparable efficacy and safety to teriparatide in the treatment of postmenopausal osteoporosis, with no significant differences observed in the risk of falls, fractures, and serious adverse events, including cardiovascular events.
- The overall quality of evidence from the systematic reviews ranged from very low to moderate, primarily due to issues of indirectness and imprecision in the outcomes assessed.
- The findings of this overview revealed substantial overlap among primary studies, with most outcomes assessed through indirect comparisons, highlighting the need for further research to confirm these results.

## 1 Introduction

Osteoporosis is a skeletal disorder characterized by low bone mass and fragility associated with an increased risk of fractures. It is a substantial public health concern, particularly impacting postmenopausal women, because of the morbidity and mortality linked with vertebral and hip fractures [1, 2]. The global prevalence of osteoporosis is 19.7% and every year, 37 million fragility fractures occur among individuals aged over 55 [3, 4]. Therefore, the primary goal of therapy in patients with osteoporosis is to reduce the risk of fractures and thereby sustain the quality of life for these individuals [5].

Different drugs are recommended for preventing fractures related to osteoporosis. Among these, bisphosphonates (e.g., alendronate, risedronate, ibandronate, and zoledronic acid) are the most widely used medicines for preventing and managing osteoporosis. However, there are concerns regarding serious adverse events, including atypical femoral fractures and osteonecrosis of the jaw as well as unproven efficacy over 5 years [6]. Moreover, therapeutic failure can occur in 25% of patients [7]. Thus, novel therapeutic agents to reduce the high risk of fractures are emerging, with variable mechanisms of action and effectiveness/safety, such as teriparatide and romosozumab [8].

Teriparatide, a recombinant human parathyroid hormone (PTH) analog, stimulates bone formation on trabecular and endocortical bone surfaces, promoting bone formation and increasing bone mineral density (BMD) [8]. In contrast, romosozumab, a monoclonal antibody targeting sclerostin, increases bone formation and reduces bone resorption, producing marked gains in spine and hip BMD [5]. Studies have demonstrated the same efficacy in reducing the high fracture risk in postmenopausal women for both agents. Although romosozumab offers better dosing convenience compared to teriparatide (monthly by subcutaneous injection for 12 months versus daily by subcutaneous injection for 24 months), their use should not be considered for women at high risk of cardiovascular disease or stroke due to associated cardiovascular risks [5, 6, 8, 9].

The increase in the availability of systematic reviews presents a challenge to healthcare professionals and decision‐makers to promote evidence‐based healthcare. Moreover, it is important to assess the methodological quality of these systematic reviews before treatment recommendations can be reliably implemented in clinical practice. Currently, there is no overview published on the use of romosozumab versus teriparatide in postmenopausal osteoporosis. Therefore, this overview aimed to summarize the evidence of the efficacy and safety of romosozumab compared to teriparatide in postmenopausal women with osteoporosis and critically appraise the methodological quality of systematic reviews.

## 2 Methods

The protocol of this systematic review was registered on the International Prospective Register of Systematic Reviews (PROSPERO number CRD42024499738). This review followed the Preferred Reporting Items for Overviews of Reviews (PRIOR) Statement [10].

### 2.1 Databases and Search Strategy

A comprehensive search for relevant literature was conducted in Medline (PubMed), Cochrane Library, Centre for Reviews and Dissemination (CRD), Embase, and Latin American and Caribbean Health Sciences Literature (LILACS) until November 28th, 2023 without data restriction and regardless of the study design and language. The gray literature was also examined for any relevant supplementary literature, using the search engine Google Scholar up to the 40 registries, excluding patents and citations, through the Publish or Perish v.8 software [11]. No language restrictions or publication dates were applied in the strategies search. The search strategies used the Medical Subject Headings (MeSH) and Emtree terms or text words related to postmenopausal osteoporosis and romosozumab. The full search strategies for all databases can be found in Table S1.

### 2.2 Study Selection

Manuscripts were considered eligible if they (1) included postmenopausal women with osteoporosis, with or without prior treatment failure; (2) assessed the effect of romosozumab compared to teriparatide; (3) reported at least one of the outcomes of interest of efficacy (vertebral fractures, non‐vertebral fractures, hip fractures, or risk of falls) and safety (serious adverse events or cardiovascular adverse events); and (4) were a systematic review of randomized clinical trials (RCT) with direct or indirect meta‐analysis. It is worth noting that the most clinically relevant outcomes for the patients were selected. Primary studies design, technical reports, conference proceedings, other types of review, a meta‐analysis performed without systematic review, and systematic reviews with meta‐analysis that included other population studies, intervention, comparator, and outcomes were excluded. The manuscripts retrieved from the databases were allocated to the Rayyan QCRI web program [12] to exclude duplicate files (Phase 1), analyze the titles and abstracts of the articles (Phase 2), and analyze complete articles whose abstracts were previously selected (Phase 3). Two reviewers (T.M.L. and T.F.G.S.B.) independently reviewed the titles and abstracts of all studies identified by the searches and discussed any discrepancies arising from consensus. When it was impossible to obtain the full text, the corresponding authors were contacted via e‐mail and the ResearchGate platform (www.researchgate.net).

### 2.3 Data Extraction

The data were collected in a preformatted spreadsheet in Microsoft Excel, including literature search and period, country, target population, intervention, and comparator with the dosage regimen, outcome measures, number of RCTs and patients included in the meta‐analysis, type of comparison of meta‐analysis, statistical model used in the meta‐analysis, effect size, heterogeneity, inconsistency, and funding source. Two independent reviewers (T.M.L. AND T.F.G.S.B.) extracted data, and disagreements were resolved by a third reviewer (G.B.G.M.). We also used the Elicit online tool (https://elicit.org/) to complement this process.

### 2.4 Methodological Quality Assessment

The methodological quality of systematic reviews was assessed using the AMSTAR‐2 (Assessing the Methodological Quality of Systematic Reviews) tool [13]. The AMSTAR‐2 consists of a 16‐item questionnaire, in which the responses are categorized as “yes,” “partial yes,” or “no.” The overall assessment was based on weaknesses in key domains (items: 2, 4, 7, 9, 11, 13, and 15) as follows: “high,” no or one non‐critical weakness; “moderate,” more than one non‐critical weakness but no critical flaws; “low,” one critical flaw with or without non‐critical weaknesses; and “critically low,” more than one critical flaw with or without non‐critical weaknesses. One investigator (T.M.L.) evaluated the studies, and a second one (T.F.G.S.B.) verified this evaluation.

### 2.5 Quality of Evidence

The quality of evidence for each outcome measured was assessed using the Grading of Recommendation Assessment, Development, and Evaluation (GRADE) tool [14]. The quality of evidence was classified into four levels: high, moderate, low, and very low, indicating confidence in the effect estimate. Initially, the quality of evidence starts as high when RCTs are analyzed. Factors regarding the risk of bias (i.e., methodological limitations), publication bias, indirectness, imprecision, and inconsistency can reduce the level of evidence of the outcome. Indirect meta‐analyses were evaluated considering the adjustments proposed by Salanti et al. [15]. The evidence profile was created from an explicit assessment of each of these factors using GRADEpro software (https://gradepro.org/). One investigator (T.F.G.S.B.) evaluated the studies, and a second investigator (T.M.L.) verified this evaluation. Any disagreement was resolved by discussion.

### 2.6 Overlap of Primary Studies

To assess the overlap of primary studies across included meta‐analyses, the Corrected Covered Area (CCA) was calculated as proposed by Pieper et al. [16], using the formula: CCA = (*N* − *r*)/[*r* × (*c* − 1)] × 100, where *N* represents the total number of primary study inclusions across all included meta‐analyses, r the number of unique primary studies, and c the number of included meta‐analyses. Results were interpreted according to the following thresholds: slight (0%–5%), moderate (6%–10%), high (11%–15%), and very high (> 15%) overlap.

Given that the included reviews may have analyzed multiple outcomes, only RCTs reporting data on the outcomes of interest defined for this overview were considered for matrix construction. Furthermore, for direct meta‐analyses, eligibility was restricted to trials that directly compared the intervention of interest (romosozumab) with a comparator of interest (teriparatide). For indirect meta‐analyses, only trials reporting at least one direct comparison of either the intervention of interest or the comparator of interest versus placebo were considered eligible.

In addition to the general citation matrix, separate citation matrices were constructed for each outcome of interest, with primary studies in rows and included meta‐analyses in columns, enabling both the calculation of outcome‐specific CCA values and the visual identification of meta‐analyses with substantial overlap.

### 2.7 Data Synthesis

The characteristics of systematic reviews, the methodological quality assessment, and the quality of evidence were provided using narrative and structured tables. The original ideas and concepts of the included studies were respected. The estimates of effect size from meta‐analysis and the confidence intervals were expressed as odds ratios (OR), relative risk (RR), or hazard ratios (HR), depending on the author's report.

## 3 Results

### 3.1 Search Results

**FIGURE 1:**
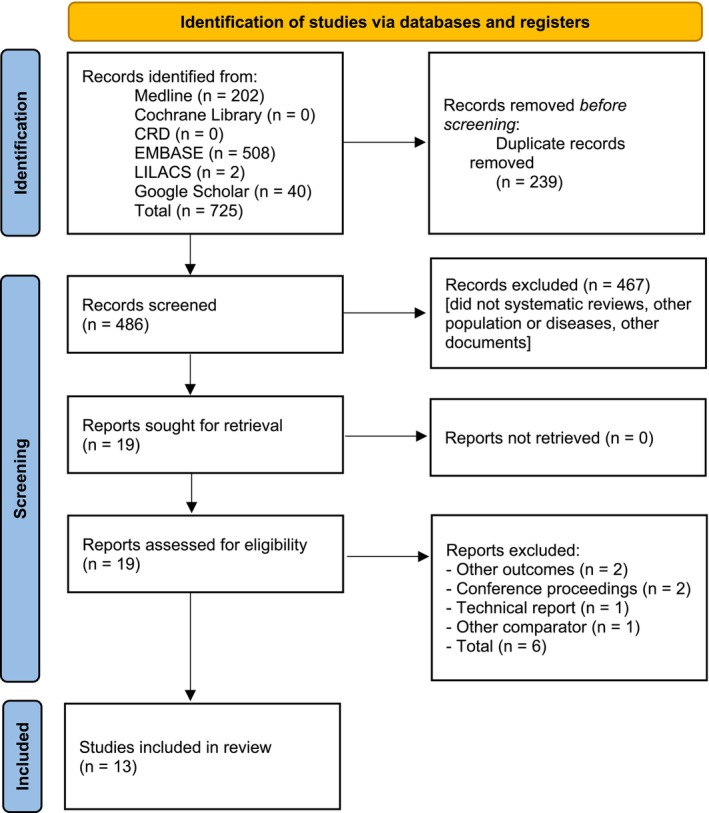
Study selection flowchart through literature search. CRD, Centre for Reviews and Dissemination.

A total of 725 potentially relevant records were retrieved from databases. After removing duplicates and screening titles and abstracts, 706 records were excluded. Full texts of the remaining 48 articles were assessed. Of these, 13 systematic reviews with meta‐analysis were included in the overview [17, 18, 19, 20, 21, 22, 23, 24, 25, 26, 27, 28, 29]. The flowchart of the literature search is shown in Figure 1. The excluded studies and the reasons for their exclusion are detailed in Table S2.

### 3.2 Characteristics of Systematic Reviews

The characteristics of the systematic reviews are summarized in Table 1. Eight reviews conducted indirect meta‐analyses and five direct meta‐analyses. All studies were published in English and were reported between 2019 and 2023, with 8 (61.5%) published in 2019 and 5 (38.5%) performed in China. Almost all reviews included primary studies evaluating only women in postmenopause with osteoporosis [17, 19, 20, 21, 22, 24, 25, 26, 27, 28, 29]. Two reviews also included patients with osteopenia [18] or low bone mass [23].

**TABLE 1:** Characteristics of systematic reviews with direct and indirect meta‐analyses included.

Authors, year	Literature search (period)	Country	Target population (*n*)	Intervention (dosage regimen)	Comparator (dosage regimen)	Outcome measure (time point)	Funding source
*Systematic reviews with direct meta‐analyses*							
Huang et al., 2023 [17]	PubMed, Cochrane Library, NIPH Clinical Trials, International Clinical Trials Registry Platform, and Clinical Trials.gov (the date of database inception to June 18, 2021)	Japan	Women in postmenopause with osteoporosis (12 796)	Romosozumab (70, 140 or 210 mg)	Teriparatide (20 μg), alendronate (70 mg), denosumab (60 mg), or placebo	Vertebral fractures (12 and 24 months), non‐vertebral fractures (12 and 24 months), clinical fractures (12 months), changes in total hip BMD (6 and 12 months), changes in lumbar spine BMD (6 and 12 months), changes in femoral neck BMD (6 and 12 months), serious AE (NR), and hypersensitivity to local administration (NR)	NR
Lv et al., 2020 [18]	PubMed, Cochrane Library, and EMBASE (the date of database inception to June 4, 2019)	China	Women in postmenopause with osteoporosis or osteopenia (85 834)	Romosozumab (NR) and denosumab (NR)	Teriparatide (NR), alendronate (NR), ibandronate (NR), risedronate (NR), or zoledronate (NR)	Cardiovascular events—3PMACE, 4PMACE (12 and 36 months)	National Key Research and Development Program
Möckel et al., 2020 [19]	PubMed, Cochrane Central Register of Controlled Trials, and ClinicalTrials.gov (the date of database inception to June 2019)	Germany	Women in postmenopause with osteoporosis (12 128)	Romosozumab (210 mg)	Teriparatide (20 μg), alendronate (70 mg), or placebo	Risk of falls (12 and 33–36 months)	NR
Singh et al., 2021 [20]	Medline (via PubMed), The Cochrane Central Register of Controlled Trials, and clinicaltrials.gov (the date of database inception to May 2020)	India	Women in postmenopause with osteoporosis (11 896)	Romosozumab (70, 140 and 210 mg)	Teriparatide (20 μg), alendronate (70 mg), denosumab (NR), or placebo	Vertebral fractures (24 months), non‐vertebral fractures (24 months), clinical fractures (24 months), risk of falls (12 and 24 months), changes in total hip BMD (12 months), changes in lumbar spine BMD (12 months), changes in femoral neck BMD (12 months), total AE (NR), and serious AE (NR)	NR
Tian et al., 2021 [21]	PubMed, Embase, the Cochrane Library, Web of Science, and the Cochrane Controlled Trials Register (the date of database inception to June 2019)	China	Women in postmenopause with osteoporosis (1304)	Romosozumab (210 mg)	Teriparatide (20 μg)	Changes in total hip BMD (6 and 12 months), changes in lumbar spine BMD (6 and 12 months), changes in femoral neck BMD (6 and 12 months), serious AE (12 months), death (12 months), and hypersensitivity to local administration (12 months)	Chinese National Natural Science Foundation, Tianjin Science and Technology, Project in the KeyField of Traditional Chinese Medicine, Tianjin Health Science and Technology Project Research, Tianjin Orthopedic Institute Foundation, Tianjin Natural Science Foundation, Tianjin Enterprise Postdoctoral Innovation Project
*Systematic reviews with indirect meta‐analyses*							
Albert et al., 2021 [22]	PubMed (January 2018 to February 2021)	United States	Women in postmenopause with osteoporosis (73 862)	Romosozumab (NR)	Teriparatide (NR), alendronate (NR), risedronate (NR), zoledronate (NR), denosumab (NR), raloxifene (NR), abaloparatide (NR), or placebo	Vertebral fractures (12, 18, 24, 36 months), hip fractures (12, 36, 60 months), and non‐vertebral fractures (12, 24, 36 months)	NR
Ayers et al., 2023 [23]	MEDLINE, Cochrane Central Register of Controlled Trials, Cochrane Database of Systematic Reviews, and ClinicalTrials.gov (January 2014 to February 2022)	United States	Women with osteoporosis or low bone mass (NR)	Romosozumab (NR)	Teriparatide (20 or 40 μg/dia, SC), alendronate (70 mg/week, or 5 or 10 mg/day, PO), ibandronate (150 mg/month, PO), risedronate (35 mg/week or 5 mg/day, PO), zoledronic acid (5 mg/year, IV), parathyroid hormone (100 μg/day, SC), abaloparatide (80 μg/day, SC), denosumab (60 mg/6 months, SC), raloxifene (60 mg/day, PO), or bazedoxifene (20 or 40 mg/day, PO)	Hip fractures (24, 36, 48 months), vertebral fractures (12, 24, 36 months), clinical fractures (12, 18, 24, 36 months), radiographic vertebral fractures (12, 24, 36, 48 months), serious AE (36 months), drug discontinuation due to serious AE (18, 36 months), atypical or subtrochanteric femoral fractures (3 to 5 years), osteonecrosis of the jaw (2 to 3 years), and atrial fibrillation (NR)	American College of Physicians
Ding et al., 2020 [24]	PubMed, Embase, and the Cochrane Central Register of Controlled Trials (the date of database inception to March 2, 2019)	China	Women in postmenopause with osteoporosis (79 144)	Romosozumab (120 mg/month, SC)	Teriparatide (20 μg/dia, SC), abaloparatide (80 μg/dia, SC), alendronate (35‐70 mg/week or 5‐10 mg/day, PO), risedronate (5 mg/day ou 35 mg/week, PO), zoledronate (5 mg/1–1,5 year, IV), denosumab (60 mg/6 months, SC), raloxifene (60 mg/day PO), ibandronate (150 mg/month, PO), parathyroid hormone (100 μg/day, SC), or placebo	Vertebral fractures (NR), non‐vertebral fractures (NR), and tolerability (NR)	NR
Seeto et al., 2023 [25]	MEDLINE and EMBASE (July 2017 to December 2020)	Australia, Canada, Ethiopian, and the United States	Women in postmenopause with osteoporosis (136 940)	Romosozumab (210 mg/month, SC)	Teriparatide (20 or 40 μg/dia, SC), abaloparatide (80 μg/day, SC), alendronate (70 mg/week), denosumab (60 mg/6 months, SC), estrogen hormones (NR), risedronate (35 mg/week or 5 mg/day, PO), ibandronate (150 mg/month, PO), or zoledronic acid (5 mg/year, IV)	Cardiovascular events—3PMACE, 4PMACE, 5PMACE (NR), heart attack (NR), stroke (NR), heart failure (NR), atrial fibrillation (NR)	National Health and Medical Research Council Australia Investigator National Heart Foundation Future Leader Amgen, Eli‐Lilly e Alexion, and honorarius of the Amgen and Sanofi
Tan et al., 2019 [26]	PubMed, Embase, the Cochrane Central Register of Controlled Trials, and GoogleScholar (the date of database inception to July 16, 2018)	China	Women in postmenopause with osteoporosis (67524)	Romosozumab (210 mg/month, SC)	Teriparatide (20 μg/dia, SC), abaloparatide (80 μg/dia, SC), denosumab (60 mg/6 months, SC), zoledronate (5 mg/year, IV), alendronate (70 mg/week or 5‐10 mg/day, PO), risedronate (5 mg/day ou 35 mg/week, PO), etidronate (400 mg/day, PO), strontium ranelate (2 g/day, PO), lasofoxifene (0.25 mg/day, PO), raloxifene (60 mg/day PO), or placebo	Vertebral fractures (NR), non‐vertebral fractures (NR), clinical fractures (NR), AE (NR), and serious AE (NR)	NR
Wei et al., 2023 [27]	PubMed, Embase, and the Cochrane Library (the date of database inception to February 15, 2020)	China	Women in postmenopause with osteoporosis (104 580)	Romosozumab (70, 140 or 210 mg)	Teriparatide (20 μg), abaloparatide(80 μg), alendronate (5‐10 mg/day or 70 mg/week), calcitonin (20 IU), denosumab (60 mg), parathyroid hormone (100 μg), risedronate (2.5 or 5 mg/day or 35 mg/week), strontium ranelate (0.5, 1 or 2 g), zoledronic acid (5 mg), raloxifene (60 mg), conjugated estrogen (0.625 mg) + medroxyprogesterone (2.5 mg), lasofoxifene (0.25 or 0.5 mg), ibandronate (150 mg), etidronate (400 mg), or bazedoxifene (20 or 40 mg)	Vertebral fractures (12 and 18 months)	Social Talent Fund of Tangdu Hospital and Tangdu Hospital Seed
Wen et al., 2020 [28]	Cochrane Library, PubMed and Embase (the date of database inception to 17 April 2019)	China	Women in postmenopause with osteoporosis (106 320)	Romosozumab (210 mg/month, SC)	Teriparatide (20 μg/dia, SC), abaloparatide (80 μg/dia, SC), denosumab (60 mg/6 months, SC), parathyroid hormone (100 μg/day, SC), zoledronate (5 mg/1‐2 year, IV), raloxifene (60 mg/day PO), alendronate (35‐70 mg/week or 5‐10 mg/day, PO), ibandronate (150 mg/month, PO), strontium ranelate (2 g/day, PO), lasofoxifene (0.5 mg/day, PO), and bazedoxifene (20 mg/day, PO)	Vertebral fractures (NR), non‐vertebral fractures (NR), tolerability (NR), and acceptability (NR)	NR
Willems et al., 2022 [29]	Medline, Medline In‐Process Citations, Epubs Ahead of Print & Daily Update, EMBASE, and Cochrane Central Register of Controlled Trials (the date of database inception to September 2, 2020)	Belgium, England, and the United States	Women in postmenopause with osteoporosis (125 165)	Romosozumab (120 mg/month, SC)	Teriparatide (20 μg/dia, SC), abaloparatide (80 μg/dia, SC), alendronate (70 mg/week or 10 mg/day, PO), risedronate (5 mg/day or 35 mg/week, PO), ibandronate (150 mg/month, PO), zoledronate (5 mg/year, IV), denosumab (60 mg/6 months, SC), or raloxifene (60 mg/day PO)	Vertebral fractures (12, 24, and 36 months), non‐vertebral fractures (12, 24, 36 months), hip fractures (12, 24, 36 months), changes in total hip BMD (NR), changes in lumbar spine BMD (NR), and changes in femoral neck BMD (NR)	UCB Pharma e Amgen Inc.

Abbreviations: 3PMACE, composite cardiovascular outcomes: death from cardiovascular causes, non‐fatal stroke, or non‐fatal myocardial infarction; 4PMACE, composite cardiovascular outcomes: 3PMACE and heart failure; 5PMACE, composite cardiovascular outcomes: 4PMACE and atrial fibrillation; AE, adverse events; BMD, bone mineral density; IV, intravenous administration; NR, not reported; PO, oral administration; SC, subcutaneous administration.

Romosozumab 210 mg was the intervention strategy most commonly used in the reviews (9; 69.2%) [17, 19, 20, 21, 25, 26, 27, 28]. Three reviews did not describe any information on the dosage regimen of the romosozumab [18, 22, 23] Almost all reviews included studies with different comparators beyond teriparatide, such as bisphosphonates, estrogen agonists/antagonists, and other PTH‐receptor agonists (abaloparatide) [17, 18, 19, 20, 22, 23, 24, 25, 26, 27, 28, 29]. Only one review included studies that used only teriparatide as the comparator [21].

Eleven reviews (84.6%) [17, 19, 20, 21, 22, 23, 24, 26, 27, 28, 29] assessed the efficacy outcomes, while nine reviews (69.2%) [18, 20, 21, 23, 24, 25, 26, 28] evaluated the safety profile of romosozumab versus teriparatide. Other outcomes also assessed in the included reviews, for example, BMD (hip, lumbar spine, or femoral neck), hypersensitivity to local administration, tolerability, and acceptability. Moreover, most reviews did not inform the time point measures for outcome assessment.

Seven studies (53.8%) [17, 19, 20, 22, 24, 26, 28] did not report a source of support for the research, five (38.5%) [18, 21, 23, 25, 27] received research funding from governments, universities, research healthcare centers, or institute organizations, and one (7.7%) [28] received research funding from the industry.

### 3.3 Results of Efficacy

Two reviews with direct meta‐analyses did not demonstrate a significant reduction in the risk of falling with treatment with romosozumab at 12 months [19] and over 12/24 months [20] in postmenopausal women when compared with teriparatide (low‐quality evidence in each). Regarding the risk of vertebral fractures, three reviews with indirect meta‐analyses did not show a significant effect of romosozumab compared to teriparatide [22, 27, 28] (low‐quality evidence). In addition, one review with indirect meta‐analysis did not report a significant difference between the two groups when analyzed over 12/24 months (RR = 0.81 [95% CI 0.19–3.34]) [23] (low‐quality evidence). The results were similar for the prevalence of vertebral fractures equal to or greater than 50% (HR = 0.79 [95% CI 0.49–1.40]) and for the prevalence of vertebral fractures less than 50% (HR = 0.98 [95% CI 0.56–1.70]) [24] (low‐quality evidence). Moreover, the findings did not show a significant difference between the two groups for the incidence of new vertebral fractures (RR = 0.84 [95% CI 0.41–1.72]) [26] and reduction of non‐vertebral fractures at 12 months (RR = 1.75 [95% CI 0.68–3.75]) [29] (low‐quality evidence).

Reviews with indirect meta‐analyses did not demonstrate a significant reduction in non‐vertebral fractures (RR = 0.91 [95% CI 0.67–1.30]) [28] (very low quality evidence) nor in their incidence (RR = 0.79 [95% CI 0.50–1.24]) [26] (very low quality evidence) in treatment with romosozumab in menopausal women when compared with teriparatide. Moreover, two reviews did not show a significant difference between the two groups when analyzed over 12 months (RR = 0.91 [95% CI 0.50–1.54]) [29] (very low quality evidence) and 12/36 months (RR = 1.38 [95% CI 0.88–2.15]) [23] (very low quality evidence).

Finally, the results of a review with indirect meta‐analysis performed between romosozumab and teriparatide for the reduction of hip fractures showed no statistical differences (*p* = 0.900) [22] (very low quality evidence). The results of efficacy are displayed in Table 2.

**TABLE 2:** Results on outcomes evaluated in systematic reviews with meta‐analyses on romosozumab compared teriparatide for postmenopausal osteoporosis.

Authors, year	Number of RCT/patients	Type of comparison	Statistical model	Pooled effect [95% CI] (*p*)	Heterogeneity *I* ^2^ (*p*)	Publication bias	Quality of evidence^a^
*Risk of falls (12/24 months)*							
Mockel et al., 2020 [19]	2/618	Direct	Random effects	RR = 1.19 [0.21–6.74] (*p* = 0.85)	*I* ^2^ = 58.5% (*p* = 0.12)	NR	Low
Singh et al., 2022 [20]	2/818	Direct	Fix effects	OR = 1.59 [0.83–3.02] (*p* = 0.16)	*I* ^2^ = 4% (*p* = 0.31)	Egger's test was not applied as studies were fewer than five	Low
*Vertebral fractures*							
Albert et al., 2023 [22]	NA	Indirect	Random effects	NR [NR] (*p* = 0.973)	NA	NR	Low
Ayers et al., 2023 [23]	NA	Indirect	Node‐splitting	RR = 0.81 [0.19–3.34] (NR)	NA	NR	Low
Ding et al., 2020 [24]	NA	Indirect	Bayesian random effects	HR = 0.79 [0.49–1.40] (NR)	NA	No asymmetry in the funnel plot	Low
Tan et al., 2019 [26]	NA	Indirect	Random effects	RR = 0.84 [0.41–1.72] (NR)	NA	NR	Low
Wei et al., 2023 [27]	NA	Indirect	Frequentist consistency	RR = 0.86 [0.38–1.96] (NR)	NA	NR	Low
Wen et al.,2020 [27]	NA	Indirect	Bayesian random effects	RR = 0.86 [0.56–1.30] (NR)	NA	No asymmetry in the funnel plot	Low
Willems et al., 2022 [28]	NA	Indirect	Bayesian random effects	RR = 1.75 [0.68–3.75]	NA	NR	Low
*Non‐vertebral fractures*							
Ayers et al., 2023 [22]	NA	Indirect	Node‐splitting	RR = 1.38 [0.88–2.15] (NR)	NA	NR	Very low
Tan et al., 2019 [25]	NA	Indirect	Random effects	RR = 0.79 [0.50–1.24] (NR)	NA	NR	Very low
Wen et al., 2020 [27]	NA	Indirect	Bayesian random effects	RR = 0.91 [0.67–1.30] (NR)	NA	No asymmetry in the funnel plot	Very low
Willems et al., 2022 [29]	NA	Indirect	Bayesian random effects	RR = 0.91 [0.50–1.54] (NR)	NA	NR	Very low
*Hip fractures*							
Albert et al., 2023 [21]	NA	Indirect	Random effects	NR [NR] (*p* = 0.900)	NA	NR	Very low
*Serious adverse events*							
Huang et al., 2023 [17]	2/537	Direct	Random effects	RR = 0.78 [0.46–1.33] (NR)	*I* ^2^ = 0% (*p* = 0.58)	No asymmetry in the funnel plot	Moderate
Tian et al., 2021 [21]	2/537	Direct	Fix effects	RR = 0.78 [0.46–1.33] (*p* = 0.37)	*I* ^2^ = 0% (*p* = 0.58)	NR	Moderate
Ayers et al., 2023 [23]	NA	Indirect	Node‐splitting	RR = 1.03 [0.70–1.51 (NR)]	NA	NR	Moderate
Tan et al., 2019 [26]	NA	Indirect	Random effects	RR = 1.07 [0.87–1.32] (NR)	NA	NR	Moderate
Wei et al., 2023 [27]	NA	Indirect	Frequentist consistency	RR = 0.97 [0.75–1.24] (NR)	NA	NR	Moderate
*Cardiovascular adverse events (3PMACE)*							
Lv et al., 2020 [18]	2/537	Direct	Fix and random effects	RR = 2.03 [0.37–11.23] (*p* = 0.42)	*I* ^2^ = 45% (NR)	No asymmetry in the funnel plot	Moderate
Seeto et al., 2023 [25]	NA	Indirect	Bayesian random effects	OR = 0.62 [0.25–1.46] (NR)	NA	NR	Moderate
*Cardiovascular adverse events (4PMACE)*							
Lv et al., 2020 [18]	2/537	Direct	Fix and random effects	RR = 1.88 [0.08–45.43] (*p* = 0.70)	*I* ^2^ = 54% (NR)	No asymmetry in the funnel plot	Moderate
Seeto et al., 2023 [25]	NA	Indirect	Bayesian random effects	OR = 0.51 [0.23–1.10] (NR)	NA	NR	Moderate

Abbreviations: 3PMACE, composite cardiovascular outcomes: death from cardiovascular causes, non‐fatal stroke, or non‐fatal myocardial infarction; 4PMACE, composite cardiovascular outcomes: 3PMACE and heart failure; NA, not applicable; NR, not reported.

^a^ The Grading of Recommendations Assessment, Development, and Evaluation (GRADE).

### 3.4 Results of the Safety Profile

Two reviews with direct meta‐analyses demonstrated no statistical differences between the romosozumab and teriparatide groups in the risk of serious adverse events (RR = 0.78 [95% CI 0.46–1.33], *p* = 0.58; *I* ^2^ = 0.0%) [17] (moderate quality evidence), nor in the 12‐month follow‐up period (RR = 0.78 [95% CI 0.46–1.33], *p* = 0.58; *I* ^2^ = 0.0%) [21] (moderate quality evidence). In addition, three reviews with indirect comparisons also did not demonstrate differences between the groups analyzed (RR = 0.97 [95% CI 0.75–1.24]) [27] (moderate quality evidence) over 12 to 36 months (RR = 1.03 [95% CI 0.70–1.51]) [23] (moderate quality evidence) or in the incidence of these adverse events (RR = 1.07 [95% CI 0.87–1.32]) [26] (moderate quality evidence).

Two reviews using direct and indirect meta‐analyses that evaluated adverse cardiovascular events (3PMACE and 4PMACE) did not observe statistically significant differences between the groups on the incidence of these outcomes in menopausal women who used romosozumab or teriparatide [18, 25] (moderate quality evidence). In the direct meta‐analysis, the adverse event 3PMACE had RR = 2.03 [95% CI 0.37–11.23] (*p* = 0.42) and 4PMACE had RR = 1.88 [95% CI 0.08–45.43] (*p* = 0.70) [18]. In indirect meta‐analyses, the adverse event 3PMACE had OR = 0.62 [95% CI 0.25–1.46] (NR), and 4PMACE had OR = 0.51 [95% CI 0.23–1.10] (NR) [25]. The safety profile results are presented in Table 2.

### 3.5 Methodological Quality of Systematic Reviews

The methodological quality of the reviews is presented in Table 3. Twelve studies (92.3%) had an “a priori” design, indicating the existence of a protocol or ethical approval [17, 18, 19, 20, 21, 23, 24, 25, 26, 27, 28, 29]. Nine studies (69.2%) conducted selection [17, 18, 20, 23, 24, 25, 27, 28, 29] and extraction [17, 18, 19, 21, 24, 25, 26, 27, 28] in duplicate, resolving differences through discussion. The majority of studies (92.3%) performed a comprehensive review of the literature, including at least two electronic databases, search strategy terms, and a supplemental search [17, 18, 19, 20, 21, 23, 24, 25, 26, 27, 28, 29]. All reviews included RCTs and described participant characteristics and interventions, and only two (15.4%) provided a list of studies excluded after full‐text evaluation [23, 27]. The quality of studies included in all systematic reviews was assessed and documented using validated tools. Ten reviews (76.9%) explained the risk of bias of individual studies when discussing the review results [17, 18, 20, 23, 24, 25, 26, 27, 28, 29] or provided a satisfactory explanation for any heterogeneity observed [17, 18, 20, 22, 23, 24, 25, 27, 28, 29]. Four reviews (30.8%) assessed the publication bias of the main outcome [17, 20, 24, 28]. All studies explicitly disclosed conflicts of interest, and seven [17, 19, 20, 22, 24, 26, 28] did not provide information on funding sources for the systematic review.

**TABLE 3:** The quality assessment results of systematic reviews with meta‐analyses included using the AMSTAR‐2 tool.

Author, year	Amstar‐2 item																Overall quality
	1	2^a^	3	4^a^	5	6	7^a^	8	9^a^	10	11^a^	12	13^a^	14	15^a^	16	
*Systematic reviews with direct meta‐analyses*																	
Huang et al., 2023 [17]	Y	Y	N	PY	Y	Y	N	Y	Y	N	Y	Y	Y	Y	Y	Y	Low
Lv et al., 2020 [18]	Y	Y	N	PY	Y	Y	N	Y	Y	N	Y	Y	Y	Y	N	Y	Low
Möckel et al., 2020 [19]	Y	PY	N	PY	N	Y	N	PY	Y	N	Y	N	N	N	N	Y	Critically low
Singh et al., 2021 [20]	Y	Y	N	PY	Y	N	N	PY	Y	N	Y	N	Y	Y	Y	Y	Low
Tian et al., 2021 [21]	Y	PY	N	PY	N	Y	N	PY	Y	N	Y	N	N	N	N	Y	Critically low
*Systematic reviews with indirect meta‐analyses*																	
Albert et al., 2021 [22]	Y	N	N	N	N	N	N	PY	PY	N	Y	N	N	Y	N	Y	Critically low
Ayers et al., 2023 [23]	Y	PY	N	PY	Y	N	PY	Y	Y	N	Y	N	Y	Y	N	Y	Low
Ding et al., 2020 [24]	Y	Y	N	PY	Y	Y	N	Y	PY	N	Y	Y	Y	Y	Y	Y	Low
Seeto et al., 2023 [25]	Y	Y	N	PY	Y	Y	N	Y	Y	N	Y	Y	Y	Y	N	Y	Critically low
Tan et al., 2019 [26]	Y	PY	N	PY	N	Y	N	PY	PY	N	Y	Y	Y	N	N	Y	Critically low
Wei et al., 2023 [27]	Y	Y	N	PY	Y	Y	Y	Y	Y	N	Y	Y	Y	Y	N	Y	Low
Wen et al., 2020 [28]	Y	Y	N	PY	Y	Y	N	Y	PY	N	Y	Y	Y	Y	Y	Y	Low
Willems et al., 2022 [29]	Y	PY	N	PY	Y	N	N	PY	Y	N	Y	Y	Y	Y	N	Y	Critically low

*Note:* Item 1: Did the research questions and inclusion criteria for the review include the components of PICO?; Item 2: Did the report of the review contain an explicit statement that the review methods were established prior to the conduct of the review and did the report justify any significant deviations from the protocol?; Item 3: Did the review authors explain their selection of the study designs for inclusion in the review?; Item 4: Did the review authors use a comprehensive literature search strategy?; Item 5: Did the review authors perform study selection in duplicate?; Item 6: Did the review authors perform data extraction in duplicate?; Item 7: Did the review authors provide a list of excluded studies and justify the exclusions?; Item 8: Did the review authors describe the included studies in adequate detail?; Item 9: Did the review authors use a satisfactory technique for assessing the risk of bias (RoB) in individual studies that were included in the review?; Item 10: Did the review authors report on the sources of funding for the studies included in the review?; Item 11: If meta‐analysis was performed did the review authors use appropriate methods for statistical combination of results?; Item 12: If meta‐analysis was performed, did the review authors assess the potential impact of RoB in individual studies on the results of the meta‐analysis or other evidence synthesis?; Item 13: Did the review authors account for RoB in individual studies when interpreting/discussing the results of the review?; Item 14: Did the review authors provide a satisfactory explanation for, and discussion of, any heterogeneity observed in the results of the review?; Item 15: If they performed quantitative synthesis did the review authors carry out an adequate investigation of publication bias (small study bias) and discuss its likely impact on the results of the review?; Item 16: Did the review authors report any potential sources of conflict of interest, including any funding they received for conducting the review?

Abbreviations: N, no; PY, partial yes; Y, yes.

^a^ Critical domains.

This overview found that seven systematic reviews had critically low overall confidence in their results [17, 18, 20, 23, 24, 27, 28], and six had low overall confidence [19, 21, 22, 25, 26, 29] according to the AMSTAR checklist.

### 3.6 Overlap of Primary Studies

Tables S3–S5 summarize the distribution of primary studies within the included reviews, limited to those that evaluated the outcomes of interest in this overview. Twelve unique primary studies were identified across 13 included meta‐analyses—five pairwise and eight network meta‐analyses—yielding an overall CCA of 22.9%, classified as very high overlap. The studies most frequently shared across reviews were Langdahl et al. [30], present in 10 of the 12 meta‐analyses (83.3%), followed by Cosman et al. [31] and Neer et al. [32], each appearing in eight meta‐analyses (66.6%), and McClung et al. [33], present in seven (58.3%). Outcome‐specific analyses revealed consistently high overlap across all evaluated outcomes. For vertebral fracture (CCA = 27.8%), the overlap was primarily driven by Cosman et al. [31] and Neer et al. [32], both of which are present in six of the seven meta‐analyses (85.7%), and Langdahl et al. [30], present in four (57.1%). For non‐vertebral fracture (CCA = 38.9%), Cosman et al. [31] and Neer et al. [32] were included in all four meta‐analyses. For serious adverse events (CCA = 32.1%), Langdahl et al. [30] appeared in four of the five meta‐analyses (80%), while McClung et al. [33] and Cosman et al. [31] were each present in three (60%). For 3P‐MACE and 4P‐MACE (CCA = 25% for both), Langdahl et al. [30] and McClung et al. [33] were the only studies shared between both meta‐analyses. Fall risk showed complete overlap (CCA = 100%), as both available meta‐analyses included the same two trials—Langdahl et al. [30] and McClung et al. [33].

## 4 Discussion

### 4.1 Main Findings

To our knowledge, this is the first overview of systematic reviews with meta‐analyses of RCTs evaluating romosozumab compared to teriparatide for postmenopausal osteoporosis. Romosozumab demonstrated efficacy and safety similar to teriparatide for all evaluated outcomes. Although 13 studies on this topic have been published since 2019, these findings were derived from systematic reviews supported by only four primary studies [30, 31, 32, 33]. Moreover, the quality of evidence for efficacy and safety main outcomes related to efficacy and safety was generally rated as moderate or low, highlighting the need for further research to improve confidence in the estimate of effects.

This overview showed that romosozumab could be an alternative and interesting option as front‐line therapy in patients with postmenopausal osteoporosis. The National Institute for Health and Care Excellence (NICE) and the Canadian Agency for Drugs and Technologies in Health (CADTH) recommended the use of romosozumab in the treatment of severe postmenopausal osteoporosis in individuals at high risk of fractures. Moreover, studies conducted in the UK [34], Belgium [35], and Japan [36] indicate that romosozumab is more cost‐effective than teriparatide in women with severe osteoporosis. Thus, it is important to consider the price agreement between the manufacturer and the healthcare system for the funding of treatment with the medicine, in addition to the existence of well‐defined clinical criteria.

Recent studies have shown the superiority of romosozumab compared to teriparatide for other efficacy outcomes, such as bone mineral density (BMD) [37, 38]. However, these results should be interpreted with caution, as these outcomes do not always translate to a lower risk of falls or fractures. Secondary outcomes are supplementary measures observed to aid in interpreting the results of the primary outcome, and they are not recommended as the central basis for decision‐making [39, 40].

This overview showed that only two systematic reviews [19, 20] with direct meta‐analysis assessed an efficacy‐related outcome—the risk of falls. Unlike conventional pairwise meta‐analysis, which is limited to comparing two interventions at a time, network meta‐analysis (NMA) enables the simultaneous comparison of multiple interventions by combining direct and indirect evidence through a common comparator [41]. However, the validity of NMA‐derived estimates—which account for most fracture‐related outcomes in this overview—depends on three core assumptions that must be critically examined: homogeneity (studies comparing the same treatments are internally comparable); transitivity (potential effect modifiers are similarly distributed across all trials contributing to the network); and consistency, also termed coherence (direct and indirect estimates for a given comparison do not differ beyond what is expected by chance) [42, 43].

Transitivity is particularly critical in the present context, as romosozumab and teriparatide were not compared head‐to‐head in the primary trials underlying these reviews; instead, indirect estimates were constructed using placebo‐controlled trials as the connecting node. This design requires that the placebo arms of romosozumab and teriparatide trials be sufficiently similar in their distribution of key effect modifiers, including baseline fracture risk, prior antiresorptive treatment, disease severity, and follow‐up duration—for indirect comparisons to be valid [44, 45]. An empirical analysis of 209 NMA networks found violations of transitivity to be common and multiple statistical tests alone to be insufficient for its detection [46]. Notably, none of the systematic reviews included in this overview reported a formal assessment of transitivity, whether through systematic tabulation of effect modifiers across contributing trials or through graphical diagnostic approaches.

Regarding consistency, although some reviews reported heterogeneity statistics within pairwise comparisons, none explicitly applied tests for global or local inconsistency, such as the decomposition of the Q‐statistic or node‐splitting analyses in frequentist frameworks or the unrelated mean effects (UME) model in Bayesian approaches [47]. The Cochrane Handbook explicitly cautions that the absence of statistically significant incoherence does not guarantee transitivity in the network [40]. Furthermore, the GRADE Working Group recommends that the certainty of each indirect estimate be rated separately, accounting for transitivity concerns, before being integrated with any available direct evidence [40, 48]. Since this step was not performed in any included review, the certainty ratings for fracture outcomes carry an additional layer of uncertainty beyond what the reported GRADE levels convey. Future research should prioritize head‐to‐head randomized trials comparing romosozumab and teriparatide directly, and future systematic reviews on this topic should explicitly report transitivity assessments and consistency tests when indirect comparisons are employed.

A significant source of heterogeneity across the included systematic reviews was variability in population characteristics. Several reviews encompassed broader populations with osteoporosis, irrespective of prior treatment history. This inconsistency may introduce clinical heterogeneity that limits the internal validity of pooled comparisons and reduces the precision of subgroup‐specific inferences [40]. To address current evidence gaps, future research should prioritize purpose‐designed trials and systematic reviews focused on specific treatment scenarios, thereby providing more reliable guidance for clinical decision‐making.

Notably, this overview revealed substantial overlap among primary studies, indicating that multiple systematic reviews synthesized largely similar evidence bases [49]. The recurrence of a select cohort of landmark trials [30, 31, 32, 33] underscores that RCT evidence remains confined to a few pivotal studies, forming the foundational basis for almost all quantitative syntheses in this domain. This pattern was more pronounced for specific outcomes: two RCTs [31, 32] contributed to all four meta‐analyses of non‐vertebral fracture, while a single RCT [30] was included in 80% of those examining serious adverse events. The complete overlap in trials assessing fall risk warrants particular caution in interpretation, as both available meta‐analyses relied on an identical set of only two trials [30, 33]. This renders their effect estimates statistically dependent and precludes independent corroboration for this outcome. Moreover, all direct meta‐analyses relied exclusively on two pivotal studies [30, 33]. Compounding this limitation, inconsistencies in effect estimates emerged even among reviews utilizing the same RCTs. It was not possible to determine whether these discrepancies arose from errors in data extraction or from reliance on secondary data sources. Consequently, these findings underscore the need for cautious interpretation, as study duplication may artificially inflate the strength of the evidence and compromise the independence of the conclusions [40].

### 4.2 Methodological Quality of Systematic Reviews

All systematic reviews included in the present overview were judged to have low or very low methodological quality according to the AMSTAR‐2 critical appraisal criteria, despite all reviews being published after the update of this tool in September 2017. These results are in line with recent overviews on the impact of traditional Chinese medicine on postmenopausal osteoporosis [50] and the role of vitamin D in preventing falls and fractures [51]. However, these results contrast with the findings of a previous overview that investigated the risks of rare serious adverse effects related to the long‐term use of bisphosphonates and applied the first version of AMSTAR [52]. The findings of this overview suggest an urgent need to improve the methodological quality of future reviews.

Among the topics needing improvement, it was noted that only 70% of the reviews conducted selection (item 5) and data extraction (item 6) in duplicate, resolving discrepancies through discussion. This finding contrasts with the studies by Chakhtoura et al. [51] and Zhao et al. [50], which showed high adherence to these items. Conducting these steps in duplicate is crucial for minimizing errors of omission of relevant studies and transcription errors of individual study data. The practice of performing these steps in duplicate is widely recommended by methodological guidelines, such as those provided by the Cochrane Handbook for Systematic Reviews of Interventions [40].

It is noteworthy that only two systematic reviews (15.4%) provided a list of excluded studies (item 7) after full‐text evaluation, which contrasts with other overviews in the field that showed higher adherence to this item [51] This information is essential for the transparency and reproducibility of the review process [53], and it can now be easily included as an electronic supplement to the article.

As observed in the overview by Zhao et al. [50], none of the analyzed systematic reviews disclosed the funding sources of the primary studies included (item 10), raising concerns about potential undisclosed conflicts of interest that could compromise the impartiality of the results. This finding is particularly troubling, as studies sponsored by the pharmaceutical industry tend to report more favorable efficacy results and conclusions than those funded by other sources [54].

Additionally, only four reviews (30.8%) assessed publication bias for the main outcome (item 15), which could distort the overall body of available evidence—a common issue in other reviews in the field [51]. This lack of assessment may be attributed to the small number of RCTs included in the evaluated reviews, as having fewer than 10 studies reduces statistical power and can lead to unreliable results [55]. However, even when statistical tests or graphical aids are not feasible, it would be beneficial to explicitly state the perceived risk of publication bias through a qualitative analysis of the search strategy and sources of evidence, for example [55].

Taken together, these findings reveal a convergent pattern of methodological deficiencies that collectively undermine the reliability of the evidence synthesized in this overview. Among the critical AMSTAR‐2 domains—those whose failure directly determines a “critically low” overall rating—the most prevalent flaws were the absence of a list of excluded studies after full‐text evaluation (item 7, absent in 84.6% of reviews) and the failure to assess publication bias for the main outcome (item 15, absent in 69.2% of reviews). A third critical domain, the failure to account for risk of bias in individual studies when interpreting the results (item 13), was also absent in 23.1% of reviews. These two critical flaws co‐occurred in the majority of reviews rated as critically low, and their combination is particularly consequential: **t**he simultaneous absence of a list of excluded studies, an assessment of publication bias, and adequate discussion of risk of bias creates a setting in which selective inclusion of favorable results cannot be ruled out. This concern is further amplified by the universal failure to disclose funding sources of the primary studies included (item 10), a non‐critical domain but one of particular epidemiological relevance, given that industry‐sponsored trials in this therapeutic area are the norm. Collectively, these deficiencies suggest that the reported effect sizes for both efficacy and safety outcomes may be subject to systematic overestimation of benefit. These limitations do not invalidate the findings of this overview, but they establish a clear ceiling on the confidence that can be placed in the current evidence base, and reinforce the need for methodologically rigorous future systematic reviews in this field.

### 4.3 Opportunities for Future Research

This overview showed that there is room to improve future studies on the use of romosozumab versus teriparatide in postmenopausal women with osteoporosis. Long‐term RCTs are essential to provide insights into the efficacy and safety of these treatments beyond the 12–24 months currently studied. In addition to fractures, important outcomes such as quality of life, pain, and mobility in these RCTs could provide a more comprehensive view of the clinical benefits of these therapies. Stratified analyses in specific subgroups of patients, such as those with different levels of disease severity or comorbid conditions, would also be valuable in revealing who might benefit most from each treatment. Finally, more RCTs on the topic with higher quality of conduct and reporting are needed to enhance the scientific evidence, especially regarding efficacy outcomes.

Future systematic reviews on the topic should be appropriately designed and conducted using a quality guide tool, such as the AMSTAR 2 checklist, as all reviews presented low or very low methodological quality. Special attention should be given to the selection and extraction of data in duplicate, the inclusion of a list of excluded studies, the declaration of funding sources for the primary studies included in the review, the explanation of the risk of bias of individual studies when discussing the review results, and the evaluation of publication bias.

### 4.4 Limitations

This overview has some limitations. Studies may have been missed because they were not indexed in the databases searched or were published on the websites of institutions or health technology assessment agencies. Moreover, the exclusion criteria applied for systematic reviews without meta‐analysis and other clinical outcomes may have led to the omission of significant reviews on this topic. In addition, we were unable to perform a quantitative analysis due to the heterogeneity among populations, interventions, and outcomes across the included reviews. Finally, the fracture‐related efficacy outcomes synthesized in this overview derive predominantly from network meta‐analyses in which none of the included reviews formally assessed the transitivity assumption—whether through systematic tabulation of effect modifiers across contributing trials or through graphical diagnostic approaches—nor applied tests for global or local statistical inconsistency (coherence). The absence of these assessments represents an additional source of uncertainty in the indirect estimates for vertebral, non‐vertebral, and hip fractures, beyond what is captured by the reported GRADE certainty ratings.

## 5 Conclusion

Thirteen systematic reviews evaluated the comparative efficacy and safety of romosozumab versus teriparatide for postmenopausal osteoporosis. Although romosozumab demonstrated effects comparable to teriparatide across key outcomes, including vertebral, non‐vertebral, and hip fractures, fall risk, and serious or cardiovascular adverse events, current evidence is insufficient to conclusively establish equivalence between these treatments. The available data are predominantly indirect, derived from reviews of low or critically low methodological quality, characterized by low to very low certainty and marked by substantial overlap among primary studies. Therefore, these findings should be interpreted with caution. Future high‐quality, head‐to‐head randomized controlled trials are required to draw definitive conclusions.

## Author Contributions

T.F.G.S.B., P.M.A., C.M.M.V., G.B.G.M., and T.M.L. had full access to all the data in the study and take responsibility for the integrity of the data and the accuracy of the data analysis. C.M.M.V., G.B.G.M., and T.M.L. designed the study. T.F.G.S.B. and T.M.L. acquired the study data. T.F.G.S.B. and T.M.L. analyzed and interpreted the data. P.M.A., G.B.G.M., and T.M.L. wrote the first draft of the manuscript. P.M.A., G.B.G.M., and T.M.L. revised the manuscript and approved it for publication.

## Funding

The authors have nothing to report.

## Ethics Statement

The authors have nothing to report.

## Conflicts of Interest

The authors declare no conflicts of interest.

## Supporting information


**Table S1:** Search strategies of the systematic review.
**Table S2:** List of excluded studies.
**Table S3:** Citation matrix of primary studies across the included systematic reviews.
**Table S4:** Corrected Covered Area (CCA) by outcome.
**Table S5:** Frequency of primary studies across outcomes.

## Data Availability

The datasets generated during and/or analyzed during this study can be obtained from the corresponding author on reasonable request.
