# Supplementary material for: Romosozumab Versus Teriparatide for the Treatment of Postmenopausal Osteoporosis: An Overview of Systematic Reviews With Direct and Indirect Meta‐Analyses
Source: Int J Rheum Dis. 2026 Apr 17;29:e70658. doi: 10.1111/1756-185x.70658 (PMC13090615; doi:10.1111/1756-185x.70658)
Supplement: Supplementary file 1 — Table S1: Search strategies of the systematic review. Table S2: List of excluded studies. Table S3: Citation matrix of primary studies across the included systematic reviews. Table S4: Corrected Covered Area (CCA) by outcome. Table S5: Frequency of primary studies across outcomes. [file APL-29-e70658-s001.docx]

Table S1. Search strategies of the systematic review

| **Database** | **Search strategy** |
| --- | --- |
| Medline | #1 "Postmenopause"[Mesh] OR Postmenopause* OR "Postmenopausal Period" OR "Post-menopausal Period" OR "Post menopausal Period" OR Post-Menopause* OR "Post Menopause*" OR "postmenopausal female" OR "postmenopausal women" OR "Osteoporosis, Postmenopausal"[Mesh] OR "Perimenopausal Bone Loss*" OR "Postmenopausal Bone Loss*" OR "Post-menopausal Bone Loss*" OR "Post-Menopausal Osteoporos*" OR "Postmenopausal Osteoporos*" OR "type 1 osteoporosis" OR "type i osteoporosis"  #2 "romosozumab"[Mesh] OR romosozumab OR AMG785 OR AMG-785 OR "AMG 785" OR “evenity” OR romosozumab-aqqg OR "romosozumab-aqqg" OR CDP7851 OR CDP-7851  #3 #1 AND #2 |
| Cochrane Library | #1 MeSH descriptor: [Postmenopause] explode all trees  #2 MeSH descriptor: [Osteoporosis, Postmenopausal] explode all trees  #3 (Postmenopause OR "Postmenopausal Period" OR "Post-menopausal Period" OR "Post menopausal Period" OR Post-Menopause OR "Post Menopause" OR "postmenopausal female" OR "postmenopausal women" OR "Perimenopausal Bone Loss" OR "Postmenopausal Bone Loss" OR "Post-menopausal Bone Loss" OR "Post-Menopausal Osteoporosis" OR "Postmenopausal Osteoporosis" OR "type 1 osteoporosis" OR "type i osteoporosis"):ti,ab,kw  #4 #1 OR #2 OR #3  #5 (romosozumab OR AMG785 OR AMG-785 OR "AMG 785" OR “evenity” OR romosozumab-aqqg OR "romosozumab-aqqg" OR CDP7851 OR CDP-7851):ti,ab,kw  #6 #4 AND #5 |
| Centre for Reviews and Dissemination (CRD) | (Postmenopausal Osteoporosis OR Postmenopause) AND (evenity OR romosozumab) |
| EMBASE | #1 'postmenopause'/exp OR postmenopause OR 'post menopause' OR 'postmenopausal female' OR 'postmenopausal period' OR 'post-menopausal period' OR 'postmenopausal women' OR 'postmenopause osteoporosis'/exp OR 'postmenopause osteoporosis' OR 'post-menopausal bone loss' OR 'post-menopausal osteoporosis' OR 'post-menopause bone loss' OR 'post-menopause osteoporosis' OR 'postmenopausal bone loss' OR 'postmenopausal osteoporosis' OR 'postmenopause bone loss' OR 'type 1 osteoporosis' OR 'type i osteoporosis'  #2 'romosozumab'/exp OR romosozumab OR 'amg 785' OR 'amg785' OR 'cdp 7851' OR 'cdp7851' OR 'evenity' OR 'romosozumab aqqg' OR romosozumab-aqqg OR 'sclerostin ab'  #3 #1 AND #2 |
| LILACS | #1 MH:"Postmenopause" OR "Postmenopause" OR "Postmenopausal Period" OR "Post-menopausal Period" OR "Post menopausal Period" OR Post-Menopause* OR "Post Menopause" OR "postmenopausal female" OR "postmenopausal women" OR MH:"Osteoporosis, Postmenopausal" OR "Perimenopausal Bone Loss*" OR "Postmenopausal Bone Loss*" OR "Post-menopausal Bone Loss*" OR "Post-Menopausal Osteoporos*" OR "Postmenopausal Osteoporos*" OR "type 1 osteoporosis" OR "type i osteoporosis"  #2 MH:"romosozumab" OR romosozumab OR AMG785 OR AMG-785 OR "AMG 785" OR “evenity” OR romosozumab-aqqg OR "romosozumab-aqqg" OR CDP7851 OR CDP-7851  #3 #1 AND #2 |
| Google Scholar | (Postmenopausal Osteoporosis OR Postmenopause) AND (evenity OR romosozumab) |

Table S2. List of excluded studies.

| **Reason for exclusion** | **Authors, year** | **Title** | **Reference** |
| --- | --- | --- | --- |
| Other outcomes | Poutoglidou et al., 2022 | Efficacy and safety of anti-sclerostin antibodies in the treatment of osteoporosis: A meta-analysis and systematic review. | J Clin Densitom. 2022;25:401-415. |
|  | Liu et al., 2018 | Romosozumab treatment in postmenopausal women with osteoporosis: a meta-analysis of randomized controlled trials. | Climacteric. 2018;21:189-195. |
| Conference Proceedings | Azharuddin et al., 2022 | Meta-analysis of effects of romosozumab on bone mineral density in postmenopausal women with osteoporosis: Evidence from randomized controlled trials. | Bone Rep. 2022;16:101472. |
|  | Selim et al., 2020 | Cardiovascular Risk in Patients Treated with Romosozumab: Disproportional Meta-Analysis. | J. Bone Miner. Res. 2020;35:246. |
| Technical report | Davis et al., 2020 | Denosumab, raloxifene, romosozumab and teriparatide to prevent osteoporotic fragility fractures: A systematic review and economic evaluation. | Health Technol Assess. 2020;24:1-314. |
| Other comparator | Händel et al., 2023 | Fracture risk reduction and safety by osteoporosis treatment compared with placebo or active comparator in postmenopausal women: systematic review, network meta-analysis, and meta-regression analysis of randomised clinical trials | BMJ. 2023;381:e068033. |

Table S3. Citation matrix of primary studies across the included systematic reviews

| **Primary Study**    **(Author, Year)** | **Direct meta-analyses** | | | | | **Indirect meta-analyses** | | | | | | | |  |
| --- | --- | --- | --- | --- | --- | --- | --- | --- | --- | --- | --- | --- | --- | --- |
|  | Huang et al., 2023 | Lv et al., 2020 | Möckel et al., 2020 | Singh et al., 2021 | Tian et al., 2021 | Albert et al., 2021 | Ayers et al., 2023 | Ding et al., 2020 | Seeto et al., 2023 | Tan et al., 2019 | Wei et al., 2023 | Wen et al., 2020 | Willems et al., 2022 | **Freq. (Σ)** |
| Langdahl et al., 2017 | 1 | 1 | 1 | 1 | 1 | — | — | 1 | 1 | 1 | 1 | 1 | — | **10** |
| McClung et al., 2014 | 1 | 1 | 1 | 1 | 1 | — | — | — | 1 | — | 1 | — | — | **7** |
| Cosman et al., 2016 | — | — | — | — | — | 1 | 1 | 1 | 1 | 1 | 1 | 1 | 1 | **8** |
| Neer et al., 2001 | — | — | — | — | — | 1 | 1 | 1 | 1 | 1 | 1 | 1 | 1 | **8** |
| Nakamura et al., 2012 | — | — | — | — | — | 1 | 1 | — | 1 | — | 1 | — | — | **4** |
| Ishibashi et al., 2017 | — | — | — | — | — | — | — | — | 1 | — | 1 | — | — | **2** |
| Fujita et al., 2014 | — | — | — | — | — | — | 1 | — | — | — | — | — | — | **1** |
| Geusens et al., 2019 | — | — | — | — | — | — | 1 | — | — | — | — | — | — | **1** |
| Miyauchi et al., 2008 | — | — | — | — | — | — | — | — | — | — | 1 | — | — | **1** |
| Lewiecki et al., 2019 | — | — | — | — | — | — | — | — | — | — | — | — | 1 | **1** |
| Greenspan et al., 2007 | — | — | — | — | — | — | — | — | 1 | — | — | — | — | **1** |
| Fogelman et al., 2008 | — | — | — | — | — | — | — | — | 1 | — | — | — | — | **1** |
| **Total per meta-analysis** | **2** | **2** | **2** | **2** | **2** | **3** | **5** | **3** | **8** | **3** | **7** | **3** | **3** | **45** |

Each cell indicates whether a primary study was included (1) or not (—) in a given meta-analysis. Shaded cells (1) denote inclusion. Freq. (Σ) = total number of meta-analyses in which each primary study appeared.

Table S4. Corrected Covered Area (CCA) by outcome.

| **Outcome** | **N** | **r** | **c** | **CCA (%)** | **Classification** |
| --- | --- | --- | --- | --- | --- |
| Overall | 45 | 12 | 13 | **21.92** | Very High (>15%) |
| Fall risk | 4 | 2 | 2 | **100.00** | Very High (>15%) |
| Vertebral fracture | 24 | 9 | 7 | **27.78** | Very High (>15%) |
| Non-vertebral fracture | 13 | 6 | 4 | **38.89** | Very High (>15%) |
| Serious adverse events | 16 | 7 | 5 | **32.14** | Very High (>15%) |
| 3P-MACE | 10 | 8 | 2 | **25.00** | Very High (>15%) |
| 4P-MACE | 10 | 8 | 2 | **25.00** | Very High (>15%) |

N = total number of primary studies in all meta-analyses; r = number of unique primary studies; c = number of included meta-analyses; CCA (%) = (N − r) / [r × (c − 1)] × 100.

Classification thresholds: slight (0–5%), moderate (6–10%), high (11–15%), very high (>15%).

Abbreviations: 3PMACE (composite cardiovascular outcomes: death from cardiovascular causes, non-fatal stroke, or non-fatal myocardial infarction), 4PMACE (composite cardiovascular outcomes: 3PMACE and heart failure).

Table S5. Frequency of primary studies across outcomes

| **Primary Study (Author, Year)** | **Overall** | **Vertebral Fracture** | **Non-Vertebral Fracture** | **Serious AEs** | **3P-MACE** | **4P-MACE** | **Fall Risk** |
| --- | --- | --- | --- | --- | --- | --- | --- |
| Langdahl et al., 2017 | 10 | 4 | 2 | 4 | 2 | 2 | 2 |
| Cosman et al., 2016 | 8 | 6 | 4 | 3 | 1 | 1 | — |
| Neer et al., 2001 | 8 | 6 | 4 | 2 | 1 | 1 | — |
| McClung et al., 2014 | 7 | — | — | 3 | 2 | 2 | 2 |
| Nakamura et al., 2012 | 5 | 3 | 1 | 2 | 1 | 1 | — |
| Ishibashi et al., 2017 | 2 | 1 | — | 1 | 1 | 1 | — |
| Fujita et al., 2014 | 1 | — | 1 | — | — | — | — |
| Geusens et al., 2019 | 1 | 1 | — | — | — | — | — |
| Miyauchi et al., 2008 | 1 | 1 | — | 1 | — | — | — |
| Lewiecki et al., 2019 | 1 | 1 | 1 | — | — | — | — |
| Greenspan et al., 2007 | 1 | — | — | — | 1 | 1 | — |
| Fogelman et al., 2008 | 1 | — | — | — | 1 | 1 | — |

Values represent the number of meta-analyses (out of the total included for each outcome) in which each primary study appeared. — indicate that the study was not included in any meta-analysis for that outcome.

Abbreviations: AEs (adverse events), MACE (major adverse cardiovascular events)
